# Rare coding *TTN* variants are associated with electrocardiographic QT interval in the general population

**DOI:** 10.1038/srep28356

**Published:** 2016-06-20

**Authors:** Ashish Kapoor, Kiranmayee Bakshy, Linda Xu, Priyanka Nandakumar, Dongwon Lee, Eric Boerwinkle, Megan L. Grove, Dan E. Arking, Aravinda Chakravarti

**Affiliations:** 1 McKusick-Nathans Institute of Genetic Medicine, Johns Hopkins University School of Medicine, Baltimore, Maryland, 21205, USA; 2 Division of Epidemiology, Human Genetics and Environmental Sciences, University of Texas Health Science Center, Houston, Texas, 77030, USA

## Abstract

We have shown previously that noncoding variants mapping around a specific set of 170 genes encoding cardiomyocyte intercalated disc (ID) proteins are more enriched for associations with QT interval than observed for genome-wide comparisons. At a false discovery rate (FDR) of 5%, we had identified 28 such ID protein-encoding genes. Here, we assessed whether coding variants at these 28 genes affect QT interval in the general population as well. We used exome sequencing in 4,469 European American (EA) and 1,880 African American (AA) ancestry individuals from the population-based ARIC (Atherosclerosis Risk In Communities) Study cohort to focus on rare (allele frequency <1%) potentially deleterious (nonsynonymous, stop-gain, splice) variants (n = 2,398 for EA; n = 1,693 for AA) and tested their effects on standardized QT interval residuals. We identified 27 nonsynonymous variants associated with QT interval (FDR 5%), 22 of which were in *TTN*. Taken together with the mapping of a QT interval GWAS locus near *TTN*, our observation of rare deleterious coding variants in *TTN* associated with QT interval show that *TTN* plays a role in regulation of cardiac electrical conductance and coupling, and is a risk factor for cardiac arrhythmias and sudden cardiac death.

Over the last decade genome wide association studies (GWAS) have been successful in identifying common variants (minor allele frequency (MAF) ≥1%), mostly non-coding, that underlie genetic variation of common diseases and quantitative traits^1^. Whole exome sequencing- (WES) and/or whole genome sequencing-(WGS) based approaches have extended such screens to include rare coding and non-coding variants (MAF < 1%)^2^. However, given the stringent thresholds set for attaining genome-wide significance (after correcting for multiple tests)^3^ and small allelic effects of individual variants^4^, the discovery of new associations has been challenging. With the number of tests being performed running in millions, correcting for the false positive rates results in a high false-negative rate due to which many true associations remain hidden. One way to increase the power to detect these true associations is to perform hypothesis-driven tests, where the search is limited to candidate genes/loci based on ancillary knowledge of genetic mapping, co-expression, protein-protein interaction, subcellular protein localization and others.

The QT interval (MIM 610141), a measurement of the duration of cardiac repolarization on an electrocardiogram (ECG), is a quantitative trait with ~30% heritability and considerable medical relevance as prolongation and shortening of the QT interval are associated with increased risk of cardiovascular morbidity and mortality^5^,^6^. Abnormal QT intervals observed in Mendelian disorders, known as long-QT syndrome and short-QT syndrome, are associated with increased risk of cardiac arrhythmias and sudden cardiac death (SCD), and are usually due to rare, high penetrance coding mutations in genes encoding ion channels and their associated proteins^7^. GWAS of the QT interval have identified at least 35 loci influencing trait variation in subjects of European American (EA) ancestry that collectively explain ~8% of the phenotypic variance in the general population^8^. Among these GWAS loci, a locus on chromosome 1q containing the gene *NOS1AP* is the major genetic regulator of QT interval and accounts for ~1% of the population trait variation^9^,^10^,^11^.

Recently, we had shown that a) an enhancer variant affecting *NOS1AP* expression is the functional basis of the observed trait association, b) over-expression of NOS1AP in cardiomyocytes leads to altered cellular electrophysiology, and c) NOS1AP is localized to cardiomyocyte intercalated discs (ID), leading us to propose that NOS1AP regulates QT interval by affecting cardiac electrical conductance and coupling^12^. Based on the localization of NOS1AP to ID we had hypothesized that ID plays a major role in regulating of QT interval and genetic variation in its components underlie the risk of cardiac arrhythmias and SCD. In support of our hypothesis, we showed that compared to genome-wide markers, common variants mapping near a specific set of 170 genes encoding ID proteins (henceforth referred to as ID genes) are significantly enriched for association with QT interval. Specifically, we identified 28 ID genes/loci, including *NOS1AP,* that showed common variants associations with QT interval (false discovery rate (FDR) 5%), many of which were missed in the GWAS and were identified by restricting to variants mapping near a functionally enriched set of ID genes^12^. In this paper, we assessed whether rare coding variants in these 28 ID genes are associated with QT interval variation as well. As opposed to GWAS that map common polymorphisms and identify positional markers and loci instead of genes, assessing association with coding variants couples the identification of functional variants with specific genes. Using variants identified in the 28 candidate ID genes by WES in 4,469 EA and 1,880 African American (AA) ancestry individuals from the ARIC^13^ (Atherosclerosis Risk in Communities) cohort, we identified multiple rare nonsynonymous variants in *TTN* associated with QT interval variation. Using *TTN*-specific functional information to annotate coding variants we observed enrichment in association signal and propose that individual protein-specific functional information will be critical in identifying disease/traits variants among a large number of variants identified by sequencing studies.

## Subjects and Methods

### Subjects

Of the 15,792 subjects in the ARIC study, we studied 5,718 EA and 2,836 AA ancestry subjects in whom we had access to GWAS^8^ and WES (unpublished) data. The ARIC study subjects and the procedures have been previously described in^13^. Briefly, ARIC is a population-based prospective cohort study of cardiovascular disease in 15,792 subjects aged 45–64 years at baseline (1987–89) who were randomly sampled from four U.S. communities (~4,000 per community). Cohort members completed four extensive clinic examinations, conducted every three years between 1987 and 1998, which included medical, social and demographic data. For assessment of QT interval at baseline, 12-lead ECG digital recording and a 2-minute paper recording of a three-lead (leads V_1_, II and V_5_) rhythm strip were made. The QT interval was determined by identifying Q-wave onset and T-wave offset in all three leads. T-wave offset was defined as the point of maximum change of slope as the T-wave merges with the baseline^5^,^14^. All individuals studied and all analyses on their samples were performed according to the Helsinki declarations, were approved by local ethics and institutional review committees of the CHARGE (Cohorts for Heart and Aging Research in Genomic Epidemiology) consortium^15^ and all participants provided informed consent.

### Library Preparation, Exome Sequencing, Variant and Genotype Calling

All the steps of library preparation, capture method, sequencing, variant and genotype calling have been detailed elsewhere^16^. All variants analyzed in this study have been submitted to Database of Single Nucleotide Polymorphisms (dbSNP)^17^, Bethesda (MD): National Center for Biotechnology Information, National Library of Medicine under the dbSNP accession: ss1998364261-ss1998377886 (dbSNP Build ID:149).

### Variant-level and Sample-level QC

WES was performed on samples from multiple cohorts as part of the CHARGE consortium^15^ and will be described elsewhere (unpublished data). In this study we analyzed only single nucleotide variants (SNVs) and ignored the small insertions/deletions (indels). Variant-level QC applied on the entire sequencing data removed variants with >20% missing data, more than 2 observed alleles, monomorphic, or with mean depth of greater than 500-fold. Then, separately within each ancestry group, variants that deviated from Hardy-Weinberg equilibrium (P < 5 × 10^−6^) were filtered out. Next, within each cohort, samples with >20% missing data were removed. We, then, assessed sequencing data from 5,718 EA and 2,836 AA ARIC subjects. In addition, we filtered out 1,249 EA and 956 AA ancestry ARIC subjects based on genetic relatedness (based on common variant genotypes^8^), genetic outliers, missing phenotype (no raw QT interval measurement), history of cardiac disease, abnormal ECG findings, or those on known QT-altering medications^13^, leaving 4,469 EA and 1,880 AA ancestry subjects for analysis.

### Variant Annotation

For each of the 28 candidate ID genes we first identified the most abundant human cardiac RefSeq^18^ transcript using RNA-seq-based gene expression data generated by the UCSD Human Reference Epigenome Mapping Project^19^ (GEO accession: GSE16256). WES variants observed were then annotated with respect to this transcript using ANNOVAR^20^,^21^. Nonsynonymous variants were further annotated with respect to the 46-species vertebrate base-wise conservation score generated using phyloP^22^ (available from the UCSC Genome Browser^23^,^24^) and overlap with a known protein domain (from the Human Protein Reference Database^25^,^26^). Nonsynonymous variants in *TTN* were also annotated with respect to the sarcomeric bands^27^. For all variants found to be associated with QT interval, we assessed the predicted functional effect using SIFT^28^ and PolyPhen-2^29^ and also looked up allele counts in the Exome Aggregation Consortium (ExAC) browser^30^.

### Statistical Analyses

We used multivariate linear regression to correct the raw QT interval measured in the ECG for known covariates: heart-rate (RR interval on ECG)^31^, age^32^ and sex^33^ as well as genotypes of common polymorphisms representing 34 QT interval GWAS hits; rs1805128 in *KCNE1* was not observed in ARIC subjects^8^. Single variant quantitative trait association analysis was performed as implemented in PLINK^34^,^35^ using a linear regression model with standardized QT interval residuals. False discovery rates were calculated from *p*-values using the Benjamini-Hochberg procedure^36^.

## Results

**Figure 1:**
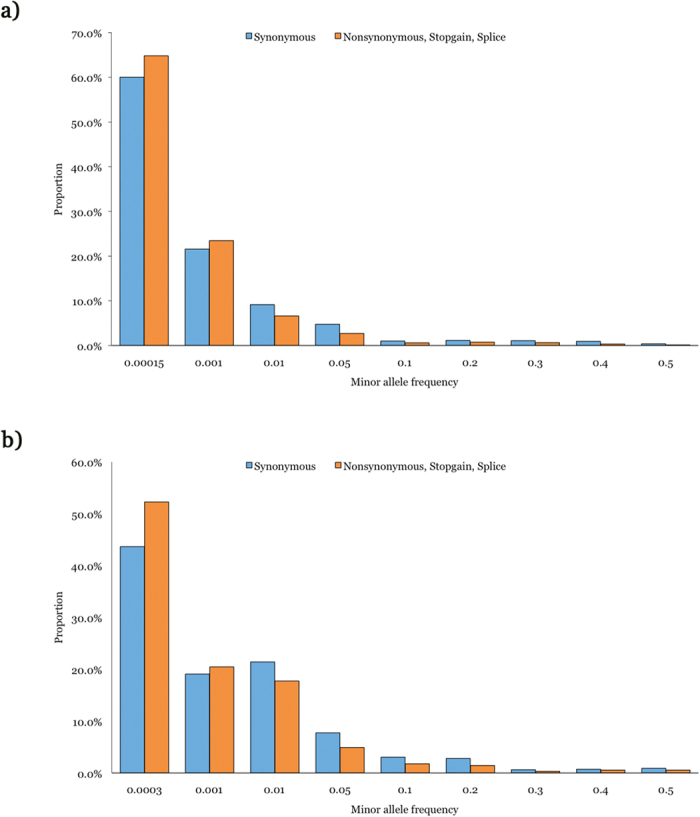
Minor allele frequency distribution of coding variants observed at 28 ID genes in EA (**a**) and AA (**b**) ARIC subjects.

Of the 5,718 and 2,836 self-identified EA and AA ARIC individuals in which WES was performed, 1,249 EA and 956 AA samples, respectively, were excluded for the following reasons a) suspected first-degree relative of an included individual based on genome-wide genotype data, b) genetic outlier based on average identity-by-state using PLINK^35^ and >8 standard deviations along any of the first 10 principal components in EIGENSTRAT^37^ after 5 iterations, c) genotypic sex not matching phenotypic sex, d) discordant with previous genotype data, e) individuals with atrial fibrillation, QRS duration >120 ms, bundle branch block or intraventricular conduction delay, f) individuals with history of heart failure or myocardial infarction, and g) when available, use of electronic pacemaker or QT-altering drugs. In the remaining 4,469 EA and 1,880 AA ARIC samples, a total of 5,878 coding SNVs (3,830 in EA subjects, 2,982 in AA subjects and overlap of 934 variants) were identified at the 28 candidate ID genes. Coding SNVs were annotated with respect to the most abundant human cardiac RefSeq transcript using ANNOVAR. Table 1 lists the 28 candidate genes, corresponding transcripts and ORF lengths, types and numbers of coding SNVs observed combined in the EA and AA ARIC subjects, and number of coding SNVs per coding base (see Supplementary Tables S1 and S2 for EA and AA only variants, respectively). A total of 2,035 synonymous, 3,777 nonsynonymous, 33 stop-gain and 33 canonical splice site SNVs were observed across all 28 ID genes. The number of coding SNVs/coding base varied from 0.017 (*KCNK3*) to 0.050 (*NRAP*) with a mean and median of 0.031, across all 28 ID genes. For each coding SNV we calculated minor allele frequency using the genotypes from the EA and AA ARIC samples separately and as expected, the majority of the coding variants were rare (Fig. 1). Of all synonymous variants, ~91% (1182/1303) in EA subjects and ~84% (935/1111) in AA subjects had allele frequency <1%, and of all nonsynonymous, stop-gain and splice variants, ~95% (2398/2527) in EA subjects and ~91% (1693/1871) in AA subjects had allele frequency of <1%. Nearly 60% of all synonymous variants and 65% of all nonsynonymous, stop-gain and splice variants were observed as singletons in EA subjects; the corresponding values for AA subjects were 44% and 52%, respectively (Fig. 1).

The raw QT interval measured in the ECG is known to be strongly dependent on heart-rate (RR interval on ECG)^31^, age^32^ and sex^33^ and needs to be corrected for these known covariates before assessing associations. Using a multivariate linear regression model, the raw QT interval measurement in 4,469 EA and 1,880 AA ARIC subjects was corrected for heart-rate, age, sex, and also for the genotypes at the 34 common GWAS variants reported to modulate QT interval in the general population (rs1805128 in *KCNE1* was not observed in ARIC subjects)^8^ (see Supplementary Figs S1 and S2 for distribution of corrected QT interval in EA and AA subjects, respectively). Of all coding SNVs identified at the 28 ID genes by WES in EA and AA ARIC subjects, we limited the association analysis to rare (MAF < 1%) and potentially deleterious (nonsynonymous, stop-gain, splice) coding variants (n = 2,398 for EA; n = 1,693 for AA). Single variant quantitative trait association analysis using a linear regression model with standardized QT residuals identified 16 and 11 variants, all nonsynonymous, in EA (Table 2) and AA (Table 3) subjects, respectively, at a FDR of 5%. Of the 16 QT interval associated variants in EA subjects 13 were in *TTN* and 1 each in *SCN5A*, *ERBB4* and *SGCZ* (Table 2); 14 variants observed as singletons in 10 subjects and 2 variants observed as doubletons in 4 subjects (Supplementary Table S3). Of the 11 QT interval associated variants in AA subjects 9 were in *TTN* and 1 each in *SCN5A* and *TLN1* (Table 3); 9 variants observed as singletons in 7 subjects and 2 variants observed as doubletons in 4 subjects (Supplementary Table S4). Thus, *TTN* had 22 out of 27 variants associated with QT interval in EA and AA subjects.

**Figure 2:**
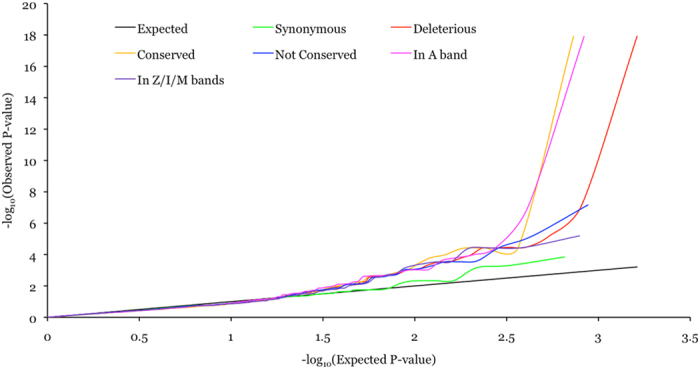
QQ plot for various classes of *TTN* variants in EA subjects.

**Figure 3:**
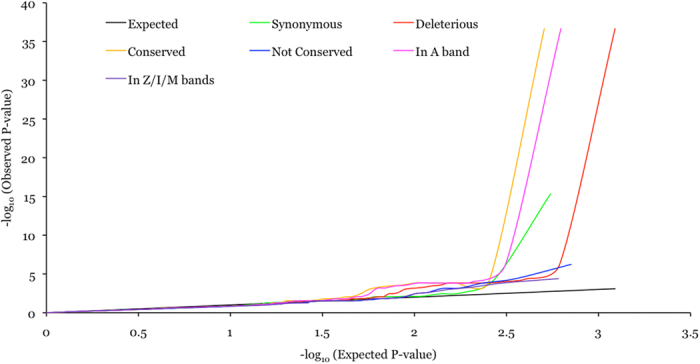
QQ plot for various classes of *TTN* variants in AA subjects.

Given that ~80% of the QT interval associated variants in this study were in *TTN* we focused on all coding variants in *TTN* and assessed whether classifying variants into various categories based on function, conservation and sarcomeric domain location, irrespective of allele frequency, might uncover additional associated variants. All *TTN* coding variants were classified by function into two classes: potentially benign (synonymous; 654/549 in EA/AA) and potentially deleterious (nonsynonymous, stop-gain and splice site; 1624/1231 in EA/AA). All nonsynonymous *TTN* variants were classified by conservation into two classes: conserved (phyloP score ≥4; 732/508 in EA/AA) and not conserved (phyloP score < 4; 877/710 in EA/AA). Titin encoded by *TTN* is the largest protein in humans and is highly expressed in striated muscle cells, where two titin molecules span each sarcomere in opposing polarity. Within the sarcomere, titin is anchored in the Z-line and spans through the I- and A-bands to the M-line. Mutations in *TTN* are a leading genetic cause of dilated cardiomyopathies (DCM) and a majority of mutations in patients are observed in the A band^27^, indicating the functional importance of the corresponding part of the protein in normal physiology. To evaluate the effect of sarcomeric domain location all potentially deleterious *TTN* variants were classified into two classes: in A band (836/625 in EA/AA) and in Z/I/M bands (788/606 in EA/AA). Quantile-quantile (QQ) plots for various classes of variants showed a trend towards association enrichment for conserved nonsynonymous variants and deleterious variants mapping to the A band (Figs 2 and 3).

## Discussion

In this study we identified multiple rare nonsynonymous variants in *TTN* associated with QT interval variation in the general population. Identification of these rare coding *TTN* variants with large genetic effects within a moderately sized subset of population-based ARIC cohort^13^, represented by 4,469 EA and 1,880 AA ancestry subjects, was made feasible by restricting association analysis of WES variants to coding variants identified in 28 ID genes, for which we had prior evidence of genetic association with QT interval based on common, mostly noncoding polymorphisms^12^. In other words, a functional-hypothesis driven approach was necessary to reduce the search space and increase the power to detect novel associations. Also, by performing association analysis based on coding variants we could directly implicate *TTN* as a causal gene, in contrast to GWAS that mostly identify positional markers. It is important to point out that only 1 out of the 28 ID genes assessed had convincing evidence for coding variants associated with QT interval, indicating that the population level trait variance is largely explained by common noncoding variants.

Titin, encoded by *TTN*, is an abundant protein of the striated muscle contractile apparatus and plays a key role in sarcomere assembly and functioning of muscle fibers. Beyond the well-documented role played by rare deleterious *TTN* mutations in causing cardiomyopathies^27^, including arrhythmogenic right ventricular dysplasia^38^,^39^, and skeletal myopathies, there is growing evidence for the role of deleterious *TTN* variants in conduction defects, in the presence or absence of cardiomyopathy and sudden cardiac death^40^,^41^. Our observations of rare coding *TTN* variants associated with QT interval variation in a population-based cohort further support a broader role of *TTN* in cardiac physiology. In addition, QT interval GWAS have mapped common noncoding variants near *TTN* at chromosome 2q31.2^8^, raising the possibility that *TTN* expression levels could also modulate cardiac repolarization.

Of the 27 rare nonsynonymous variants associated with QT interval in EA and AA ancestry ARIC subjects, 22 were in *TTN*, 2 in *SCN5A* and 1 each in *ERBB4*, *SGCZ* and *TLN1*. Among these 5 genes, based on the number of associated variants uncovered, we conclude that *TTN* has the potential to regulate QT interval variation. Although, the role of *SCN5A* in regulating QT interval is well established, based on genetic studies of rare Mendelian long-QT syndrome^7^, we observed only 2 variants in *SCN5A* associated with QT interval variation in the general population. Further association studies would be necessary to assess whether variation in the remaining 3 genes, *ERBB4*, *SGCZ* and *TLN1*, regulates QT interval in the general population. A major limitation of our study is that the 27 QT interval associated variants identified were observed as singletons (23 variants) or doubletons (4 variants) (see Supplementary Table S5 for allele counts in different populations), which raises the possibility that some of these could be false-positives. Therefore, performing similar association studies in independent and larger cohorts is, as always, necessary. Another important question that remains to be explored is whether and how these variants affect gene function (see Supplementary Table S6 for predicted functional effect from SIFT^28^ and PolyPhen-2^29^). In summary, using a hypothesis-based approach to limit association analysis of WES variants to coding variants in 28 ID genes, we identified multiple rare nonsynonymous *TTN* variants associated with QT interval variation in the general population, providing evidence of a new role for *TTN* in cardiac electrical conduction and coupling.

## Additional Information

**How to cite this article**: Kapoor, A. *et al.* Rare coding *TTN* variants are associated with electrocardiographic QT interval in the general population. *Sci. Rep.* **6**, 28356; doi: 10.1038/srep28356 (2016).

## Supplementary Material

Supplementary Information

## Figures and Tables

**Table 1:** Gene symbol, most abundant cardiac transcript, ORF length, number and type of coding variants observed in EA and AA ARIC subjects, and number of coding variants per coding base for the 28 ID genes.

Gene	Transcript^1^	ORF length	Synonymous	Nonsynonymous	Stopgain	Splice	All	#Variants/ORF length
*ANKRD30A*	NM_052997	4026	42	106	3	3	154	0.038
*ATP1B1*	NM_001677	912	15	3	0	0	18	0.020
*CAV1*	NM_001172896	444	4	11	0	0	15	0.034
*CAV2*	NM_001233	489	3	10	0	0	13	0.027
*CD59*	NM_001127223	387	9	5	0	0	14	0.036
*CDH11*	NM_001797	2391	34	45	0	0	79	0.033
*CDH2*	NM_001792	2721	32	42	0	0	74	0.027
*CIB1*	NM_001277764	696	10	11	1	0	22	0.032
*ERBB4*	NM_005235	3927	35	53	1	1	90	0.023
*KCNJ4*	NM_152868	1338	27	8	0	0	35	0.026
*KCNK3*	NM_002246	1185	12	8	0	0	20	0.017
*LRFN2*	NM_020737	2370	46	32	0	0	78	0.033
*NOS1AP*	NM_014697	1521	24	18	0	0	42	0.028
*NRAP*	NM_001261463	5196	76	171	7	8	262	0.050
*PARVA*	NM_018222	1239	21	21	0	2	44	0.036
*PKP2*	NM_004572	2646	25	63	1	2	91	0.034
*PKP4*	NM_003628	3579	45	73	1	2	121	0.034
*PRKCA*	NM_002737	2019	28	22	1	0	51	0.025
*PTK2*	NM_001199649	3198	28	47	1	1	77	0.024
*SCN5A*	NM_000335	6048	109	130	2	0	241	0.040
*SGCZ*	NM_139167	939	6	32	1	1	40	0.043
*SIPA1L1*	NM_001284247	5412	66	88	0	0	154	0.028
*SLC16A1*	NM_003051	1503	13	20	0	0	33	0.022
*SLC4A1*	NM_000342	2736	44	83	0	0	127	0.046
*SLC8A1*	NM_021097	2922	35	45	0	0	80	0.027
*SPTBN1*	NM_003128	7095	127	87	0	0	214	0.030
*TLN1*	NM_006289	7626	95	95	0	0	190	0.025
*TTN*	NM_001267550	107976	1024	2448	14	13	3499	0.032
			2035	3777	33	33	5878	

^1^Most abundant human cardiac transcript.

**Table 2:** Coding variants associated with QT interval in EA ARIC subjects.

Chr:Position (hg19)	Gene	cDNA change	Protein change	MAF	Beta	P	PhyloP score	Protein domain	ExAC allele count
2:179455718	*TTN*	c.G60734A	p.R20245Q	0.00011	8.78	1.22 × 10^−18^	6.39	FN2	4/120604
2:179407497	*TTN*	c.G97084T	p.A32362S	0.00011	5.39	6.89 × 10^−8^	0.15	–	–
2:212522534	*ERBB4*	c.C1891T	p.H631Y	0.00022	3.69	1.73 × 10^−7^	4.15	–	–
2:179634919	*TTN*	c.A8509T	p.S2837C	0.00022	3.19	6.43 × 10^−6^	3.73	IG	12/121362
2:179473995	*TTN*	c.A52042G	p.M17348V	0.00011	4.14	3.33 × 10^−5^	1.38	FN3	2/96388
2:179398282	*TTN*	c.C103060T	p.P34354S	0.00011	4.14	3.47 × 10^−5^	5.98	–	–
2:179629385	*TTN*	c.A9857G	p.K3286R	0.00011	4.11	3.81 × 10^−5^	5.11	IGC2	15/121360
2:179496930	*TTN*	c.C43691G	p.S14564C	0.00011	4.11	3.98 × 10^−5^	6.35	–	5/81630
2:179466803	*TTN*	c.C55195T	p.P18399S	0.00011	3.88	0.00010	4.14	FN3	1/120260
8:13959964	*SGCZ*	c.G665T	p.G222V	0.00011	3.78	0.00015	5.48	–	–
2:179424880	*TTN*	c.T85979C	p.I28660T	0.00011	3.77	0.00016	5.31	IGC2	2/120508
2:179413763	*TTN*	c.G92590A	p.D30864N	0.00011	3.66	0.00025	3.76	FN3	16/120638
2:179486250	*TTN*	c.A45301C	p.N15101H	0.00011	3.62	0.00029	3.60	–	–
2:179644182	*TTN*	c.A3737T	p.H1246L	0.00011	3.63	0.00029	3.43	–	–
3:38592534	*SCN5A*	c.G5326A	p.V1776M	0.00011	3.62	0.00029	5.80	–	3/121080
2:179666975	*TTN*	c.G185A	p.R62H	0.00011	3.56	0.00037	6.21	IGC2	15/121364

**Table 3:** Coding variants associated with QT interval in AA ARIC subjects.

Chr:Position (hg19)	Gene	cDNA change	Protein change	MAF	Beta	P	PhyloP score	Protein domain	ExAC allele count
9:35711334	*TLN1*	c.A3937G	p.S1313G	0.00026	15.81	3.60 × 10^−60^	3.48	–	2/121374
2:179455331	*TTN*	c.C61121T	p.P20374L	0.00053	8.85	2.26 × 10^−37^	6.39	–	2/119910
2:179447784	*TTN*	c.C65746T	p.R21916W	0.00026	4.98	5.87 × 10^−07^	0.65	IGC2	13/113940
3:38645514	*SCN5A*	c.G1579A	p.G527R	0.00026	4.33	1.48 × 10^−05^	3.93	–	4/90200
2:179528396	*TTN*	c.C36490A	p.P12164T	0.00026	4.10	4.03 × 10^−05^	−0.49	IG	7/117870
2:179447313	*TTN*	c.C65870T	p.P21957L	0.00026	3.90	9.92 × 10^−05^	6.22	FN3	–
2:179428672–179428673	*TTN*	c.82186_82187CA > GT	p.Q27396V	0.00026	3.79	0.00015	4.74, 5.31	FN3	1/120622, 1/120618
2:179451505	*TTN*	c.G64123A	p.V21375M	0.00026	3.79	0.00015	6.37	FN3	1/120562
2:179497341	*TTN*	c.G43392A	p.M14464I	0.00026	3.79	0.00015	1.68	IGC2	3/120562
2:179434555	*TTN*	c.G76304A	p.C25435Y	0.00026	3.62	0.00029	4.48	IGC2	1/120440
2:179440480	*TTN*	c.T70379G	p.L23460R	0.00053	2.52	0.00036	5.13	FN3	–
