# Supplementary material for: Rare coding TTN variants are associated with electrocardiographic QT interval in the general population
Source: Sci Rep. 2016 Jun 20;6:28356. doi: 10.1038/srep28356 (PMC4913250; doi:10.1038/srep28356)

Rare coding *TTN* variants are associated with electrocardiographic QT interval in the general population

Ashish Kapoor[1#], Kiranmayee Bakshy[1#], Linda Xu[1], Priyanka Nandakumar[1], Dongwon Lee[1], Eric Boerwinkle[2], Megan L. Grove[2], Dan E. Arking[1], Aravinda Chakravarti[1*]

Supplementary Table S1: Coding variants identified in 4,469 EA subjects in 28 ID genes.

Gene	Transcript[1]	ORF length	Synonymous	Nonsynonymous	Stopgain	Splice	All	#Variants/ ORF length
ANKRD30A	NM_052997	4026	29	69	2	3	103	0.026
ATP1B1	NM_001677	912	10	2	0	0	12	0.013
CAV1	NM_001172896	444	2	7	0	0	9	0.020
CAV2	NM_001233	489	2	7	0	0	9	0.018
CD59	NM_001127223	387	6	2	0	0	8	0.021
CDH11	NM_001797	2391	22	30	0	0	52	0.022
CDH2	NM_001792	2721	21	25	0	0	46	0.017
CIB1	NM_001277764	696	3	5	0	0	8	0.011
ERBB4	NM_005235	3927	25	36	1	0	62	0.016
KCNJ4	NM_152868	1338	19	7	0	0	26	0.019
KCNK3	NM_002246	1185	5	6	0	0	11	0.009
LRFN2	NM_020737	2370	28	24	0	0	52	0.022
NOS1AP	NM_014697	1521	14	12	0	0	26	0.017
NRAP	NM_001261463	5196	54	119	3	3	179	0.034
PARVA	NM_018222	1239	15	15	0	1	31	0.025
PKP2	NM_004572	2646	10	39	0	1	50	0.019
PKP4	NM_003628	3579	27	50	0	0	77	0.022
PRKCA	NM_002737	2019	15	14	1	0	30	0.015
PTK2	NM_001199649	3198	17	32	0	1	50	0.016
SCN5A	NM_000335	6048	63	79	1	0	143	0.024
SGCZ	NM_139167	939	5	22	1	0	28	0.030
SIPA1L1	NM_001284247	5412	46	63	0	0	109	0.020
SLC16A1	NM_003051	1503	7	12	0	0	19	0.013
SLC4A1	NM_000342	2736	29	55	0	0	84	0.031
SLC8A1	NM_021097	2922	27	26	0	0	53	0.018
SPTBN1	NM_003128	7095	86	63	0	0	149	0.021
TLN1	NM_006289	7626	62	64	0	0	126	0.017
TTN	NM_001267550	107976	654	1609	9	6	2278	0.021
			1303	2494	18	15	3830	

[1]Most abundant human cardiac transcript

Supplementary Table S2: Coding variants identified in 1,880 AA subjects in 28 ID genes.

Gene	Transcript[1]	ORF length	Synonymous	Nonsynonymous	Stopgain	Splice	All	#Variants/ ORF length
ANKRD30A	NM_052997	4026	21	58	2	1	82	0.020
ATP1B1	NM_001677	912	8	1	0	0	9	0.010
CAV1	NM_001172896	444	3	4	0	0	7	0.016
CAV2	NM_001233	489	2	5	0	0	7	0.014
CD59	NM_001127223	387	6	3	0	0	9	0.023
CDH11	NM_001797	2391	17	23	0	0	40	0.017
CDH2	NM_001792	2721	17	23	0	0	40	0.015
CIB1	NM_001277764	696	9	7	1	0	17	0.024
ERBB4	NM_005235	3927	18	19	0	1	38	0.010
KCNJ4	NM_152868	1338	16	1	0	0	17	0.013
KCNK3	NM_002246	1185	9	3	0	0	12	0.010
LRFN2	NM_020737	2370	29	13	0	0	42	0.018
NOS1AP	NM_014697	1521	14	8	0	0	22	0.014
NRAP	NM_001261463	5196	41	92	4	5	142	0.027
PARVA	NM_018222	1239	10	8	0	1	19	0.015
PKP2	NM_004572	2646	16	34	1	1	52	0.020
PKP4	NM_003628	3579	24	31	1	2	58	0.016
PRKCA	NM_002737	2019	17	12	0	0	29	0.014
PTK2	NM_001199649	3198	19	15	1	0	35	0.011
SCN5A	NM_000335	6048	70	69	1	0	140	0.023
SGCZ	NM_139167	939	1	14	0	1	16	0.017
SIPA1L1	NM_001284247	5412	37	36	0	0	73	0.013
SLC16A1	NM_003051	1503	8	10	0	0	18	0.012
SLC4A1	NM_000342	2736	24	41	0	0	65	0.024
SLC8A1	NM_021097	2922	11	21	0	0	32	0.011
SPTBN1	NM_003128	7095	63	31	0	0	94	0.013
TLN1	NM_006289	7626	52	35	0	0	87	0.011
TTN	NM_001267550	107976	549	1218	5	8	1780	0.016
			1111	1835	16	20	2982	

[1]Most abundant human cardiac transcript

Supplementary Table S3: Sample ID(s) and corrected QT interval(s) of EA subjects carrying the QT interval associated variants.

Chr.Position (hg19)	Sample ID	Corrected QT Interval (ms)	Sample ID	Corrected QT Interval (ms)
2:179455718	13	530.48		
2:179407497	16	479.77		
2:212522534	13	530.48	1	378.04
2:179634919	8	473.63	14	419.94
2:179473995	11	461.01		
2:179398282	11	461.01		
2:179629385	10	460.66		
2:179496930	10	460.66		
2:179466803	3	457.08		
8:13959964	6	455.62		
2:179424880	6	455.62		
2:179413763	21	453.91		
2:179486250	15	453.33		
2:179644182	9	453.27		
3:38592534	15	453.33		
2:179666975	19	452.35		

Supplementary Table S4: Sample ID(s) and corrected QT interval(s) of AA subjects carrying the QT interval associated variants.

Chr.Position (hg19)	Sample ID	Corrected QT Interval (ms)	Sample ID	Corrected QT Interval (ms)
9:35711334	22	694.91		
2:179455331	22	694.91	2	436.01
2:179447784	20	493.94		
3:38645514	12	481.65		
2:179528396	4	477.34		
2:179447313	5	473.02		
2:179428672-179428673	17	471.48		
2:179451505	17	471.48		
2:179497341	17	471.48		
2:179434555	7	468.33		
2:179440480	5	473.02	18	422.24

Supplementary Table S5: Allele counts from distinct ethnicities from the ExAC browser for the QT interval associated variants.

Chr:Position (hg19)	Gene	cDNA change	Protein change	dbSNP ID	ExAC allele count	European (Non-Finnish)	European (Finnish)	African	Latino	South Asian	East Asian	Other
2:179398282	*TTN*	c.C103060T	p.P34354S	-	-							
2:179407497	*TTN*	c.G97084T	p.A32362S	-	-							
2:179413763	*TTN*	c.G92590A	p.D30864N	rs200621611	16/120638	10/66694	2/6608	0/9798	2/11542	2/16510	0/8588	0/898
2:179424880	*TTN*	c.T85979C	p.I28660T	rs397517733	2/120508	0/66620	0/6612	0/9778	0/11542	2/16496	0/8562	0/898
2:179428672-179428673	*TTN*	c.82186_82187CA>GT	p.Q27396V	rs367804525, rs371604558	1/120622, 1/120618	1/66700, 1/66700	0/6610, 0/6610	0/9790, 0/9792	0/11538, 0/11536	0/16510, 0/16510	0/8570, 0/8574	0/900, 0/900
2:179434555	*TTN*	c.G76304A	p.C25435Y	rs767496380	1/120440	1/66610	0/6590	0/9792	0/11518	0/16500	0/8534	0/896
2:179440480	*TTN*	c.T70379G	p.L23460R	-	-							
2:179447313	*TTN*	c.C65870T	p.P21957L	-	-							
2:179447784	*TTN*	c.C65746T	p.R21916W	rs200155485	13/113940	9/63072	0/6386	1/9364	2/10772	1/15842	0/7642	0/862
2:179451505	*TTN*	c.G64123A	p.V21375M	rs371670651	1/120562	1/66702	0/6612	0/9798	0/11526	0/16510	0/8514	0/900
2:179455331	*TTN*	c.C61121T	p.P20374L	rs372969669	2/119910	0/66430	0/6612	2/9686	0/11522	0/16236	0/8530	0/894
2:179455718	*TTN*	c.G60734A	p.R20245Q	rs575837567	4/120604	0/66684	0/6614	2/9784	1/11548	0/16502	1/8572	0/900
2:179466803	*TTN*	c.C55195T	p.P18399S	rs774591174	1/120260	1/66594	0/6596	0/9790	0/11472	0/16480	0/8432	0/896
2:179473995	*TTN*	c.A52042G	p.M17348V	rs780570190	2/96388	0/56756	0/6604	0/8364	0/9774	0/7516	2/7236	0/698
2:179486250	*TTN*	c.A45301C	p.N15101H	-	-							
2:179496930	*TTN*	c.C43691G	p.S14564C	rs377015571	5/81630	5/44002	0/4888	0/7184	0/7190	0/11994	0/5730	0/642
2:179497341	*TTN*	c.G43392A	p.M14464I	rs370601384	3/120562	1/66652	0/6612	0/9780	1/11562	0/16506	0/8552	1/898
2:179528396	*TTN*	c.C36490A	p.P12164T	rs373422655	7/117870	0/64676	0/6614	6/8986	1/11572	0/16508	0/8622	0/892
2:179629385	*TTN*	c.A9857G	p.K3286R	rs200052398	15/121360	12/66732	0/6614	2/10406	0/11562	1/16512	0/8626	0/908
2:179634919	*TTN*	c.A8509T	p.S2837C	rs202024134	12/121362	11/66734	1/6614	0/10396	0/11558	0/16512	0/8640	0/908
2:179644182	*TTN*	c.A3737T	p.H1246L	rs201988645	-							
2:179666975	*TTN*	c.G185A	p.R62H	rs758169489	15/121364	2/66738	0/6614	0/10406	0/11574	2/16512	11/8612	0/908
2:212522534	*ERBB4*	c.C1891T	p.H631Y	-	-							
3:38592534	*SCN5A*	c.G5326A	p.V1776M	rs199473314	3/121080	1/66740	0/6614	0/10076	0/11578	1/16512	1/8652	0/908
3:38645514	*SCN5A*	c.G1579A	p.G527R	rs763550164	4/90200	1/50462	0/4766	0/7414	1/7450	1/13184	1/6260	0/664
8:13959964	*SGCZ*	c.G665T	p.G222V	rs769233485	-							
9:35711334	*TLN1*	c.A3937G	p.S1313G	rs762642967	2/121374	1/66714	0/6614	0/10402	1/11574	0/16512	0/8650	0/908

Supplementary Table S6: Predicted functional effect from SIFT and PolyPhen-2 for the QT interval associated variants.

**Chr:Position (hg19)**	**Gene**	**cDNA change**	**Protein change**	**dbSNP ID**	**SIFT score**	**SIFT prediction**	**PolyPhen-2 score**	**PolyPhen-2 prediction**
2:179398282	*TTN*	c.C103060T	p.P34354S	-	0.093	Tolerated	0.857	Possibly damaging
2:179407497	*TTN*	c.G97084T	p.A32362S	-	0.613	Tolerated	0.001	Benign
2:179413763	*TTN*	c.G92590A	p.D30864N	rs200621611	0.137	Tolerated	0.873	Possibly damaging
2:179424880	*TTN*	c.T85979C	p.I28660T	rs397517733	0.008	Deleterious	0.201	Benign
2:179428672-179428673	*TTN*	c.82186_82187CA>GT	p.Q27396V	rs367804525, rs371604558	0.094, 0.681	Tolerated, Tolerated	0.473, 0.425	Possibly damaging, Benign
2:179434555	*TTN*	c.G76304A	p.C25435Y	rs767496380	0.005	Deleterious	0.879	Possibly damaging
2:179440480	*TTN*	c.T70379G	p.L23460R	-	0.117	Tolerated	0.995	Probably damaging
2:179447313	*TTN*	c.C65870T	p.P21957L	-	0.002	Deleterious	1	Probably damaging
2:179447784	*TTN*	c.C65746T	p.R21916W	rs200155485	0.005	Deleterious	1	Probably damaging
2:179451505	*TTN*	c.G64123A	p.V21375M	rs371670651	0.005	Deleterious	1	Probably damaging
2:179455331	*TTN*	c.C61121T	p.P20374L	rs372969669	0.138	Tolerated	1	Probably damaging
2:179455718	*TTN*	c.G60734A	p.R20245Q	rs575837567	0.018	Deleterious	1	Probably damaging
2:179466803	*TTN*	c.C55195T	p.P18399S	rs774591174	0.424	Tolerated	0.987	Probably damaging
2:179473995	*TTN*	c.A52042G	p.M17348V	rs780570190	0.503	Tolerated	0	Benign
2:179486250	*TTN*	c.A45301C	p.N15101H	-	0.333	Tolerated	0.009	Benign
2:179496930	*TTN*	c.C43691G	p.S14564C	rs377015571	0.009	Deleterious	1	Probably damaging
2:179497341	*TTN*	c.G43392A	p.M14464I	rs370601384	0.022	Deleterious	0.002	Benign
2:179528396	*TTN*	c.C36490A	p.P12164T	rs373422655	.	.	0.02	Benign
2:179629385	*TTN*	c.A9857G	p.K3286R	rs200052398	0.22	Tolerated	1	Probably damaging
2:179634919	*TTN*	c.A8509T	p.S2837C	rs202024134	0.006	Deleterious	1	Probably damaging
2:179644182	*TTN*	c.A3737T	p.H1246L	rs201988645	0.63	Tolerated	0.2	Benign
2:179666975	*TTN*	c.G185A	p.R62H	rs758169489	0.347	Tolerated	1	Probably damaging
2:212522534	*ERBB4*	c.C1891T	p.H631Y	-	0.053	Tolerated	0	Benign
3:38592534	*SCN5A*	c.G5326A	p.V1776M	rs199473314	0	Deleterious	1	Probably damaging
3:38645514	*SCN5A*	c.G1579A	p.G527R	rs763550164	0.008	Deleterious	1	Probably damaging
8:13959964	*SGCZ*	c.G665T	p.G222V	rs769233485	0.004	Deleterious	1	Probably damaging
9:35711334	*TLN1*	c.A3937G	p.S1313G	rs762642967	0.168	Tolerated	0.104	Benign

Supplementary Figure S1: Corrected QT interval distribution in 4,469 EA subjects.

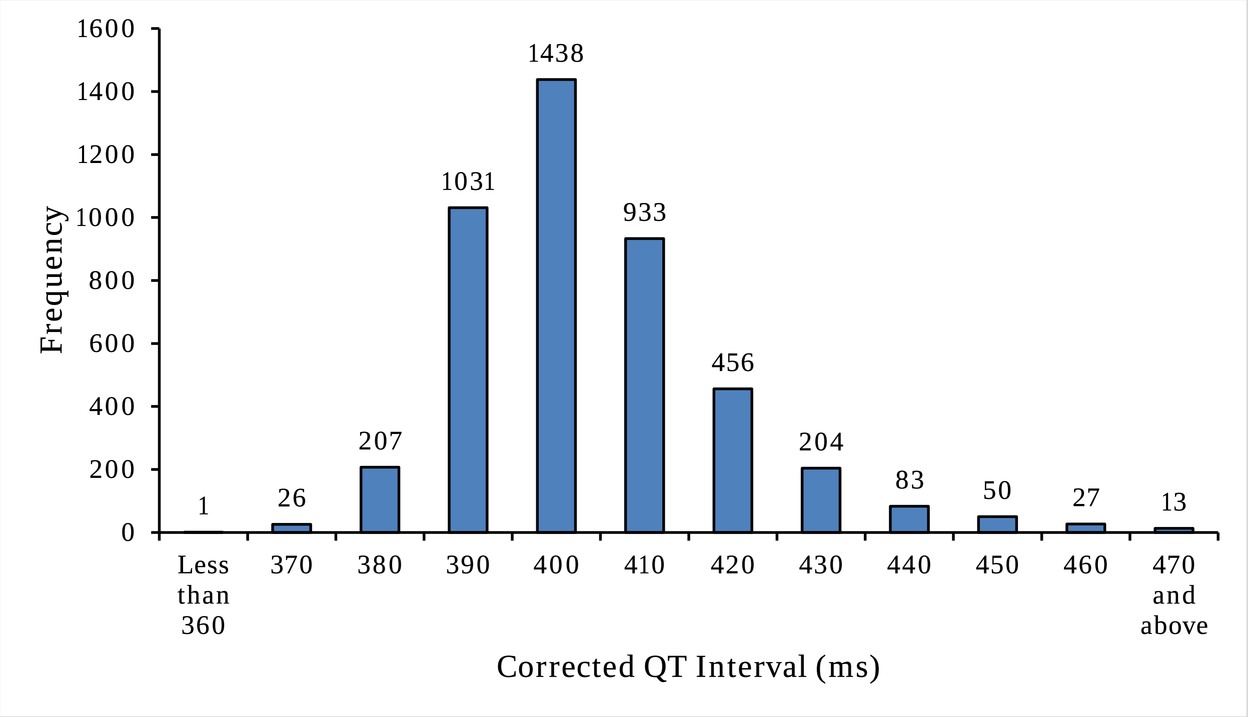

Supplementary Figure S2: Corrected QT interval distribution in 1,880 AA subjects.

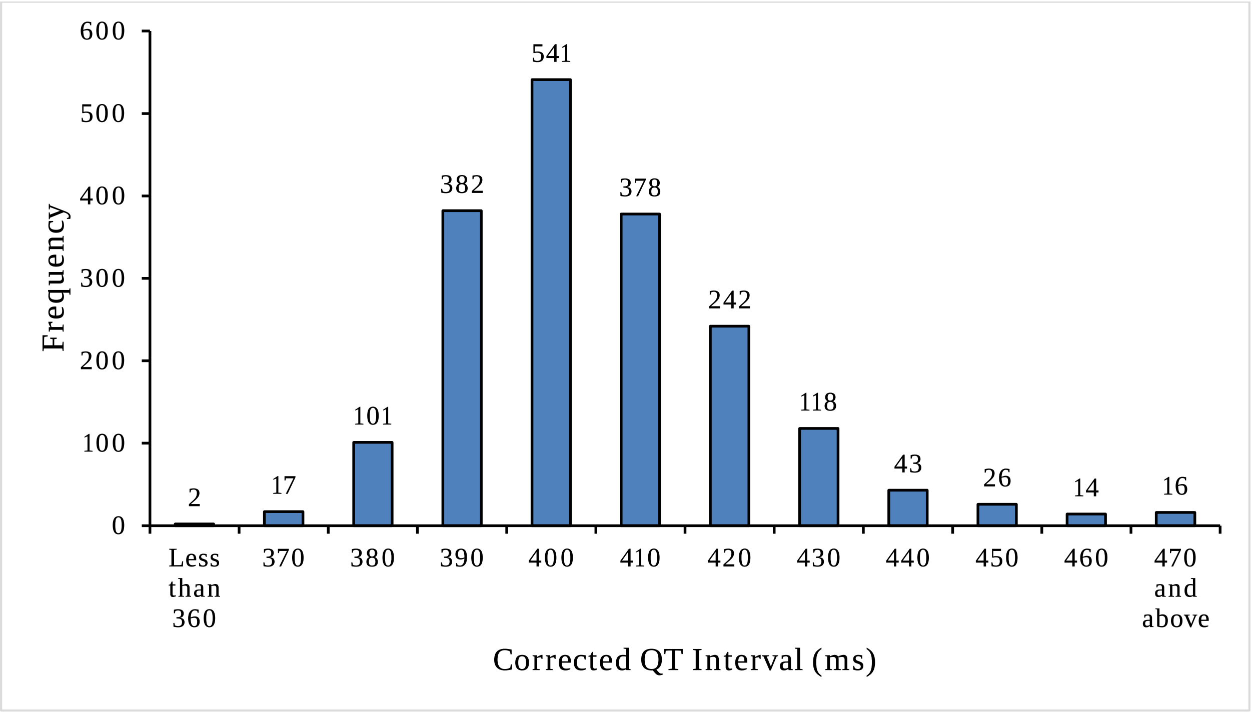

Supplement: Supplementary Information [file srep28356-s1.pdf]
